# An automated sampler for high temperature headspace gas chromatography

**DOI:** 10.1155/S1463924678000073

**Published:** 1978

**Authors:** J. B. Pausch, R. E. Arner, R. P. Lattimer

**Affiliations:** The B F Goodrich Research & Development Center 9921 Brecksville Road Brecksville Ohio 44141 USA


### An automated sampler for high temperature headspace gas chromatography

**J. B. Pausch, R. E. Arner and R. P. Lattimer**

*The B F Goodrich Research & Development Center, 9921 Brecksville Road, Brecksville, Ohio 44141, USA*

**The** environmental concern for chemicals covers a broad spectrum. One of the specific concerns in the chemical industry today is the discharge of residual volatile chemicals (normally solvents, monomers, and impurities) from commercial polymeric materials into the atmosphere. The polymer manufacturing worker, the material processing and fabricating workers, and finally the consumer, all must be protected from exposure to toxic substances, undesirable odours, or any other type of irritant that may result from undesirable volatiles or decomposition products of polymeric materials. Modern requirements dictate that these chemicals be measured at parts-per-million (ppm) levels or lower.

For many years the conventional analytical instrument for measuring residual monomers, solvents, and other small-molecule impurities in polymers has been the gas chromatograph (GC). Traditional sample preparation involves the use of solvent either to dissolve the polymer completely or to extract low molecular weight material from the polymer. The resulting solution is then chromatographed. The inherent disadvantages of either solvent extractor or solution methods can be summarised as follows: First, extensive sample preparation time is involved. Dissolution or extraction of the sample may require several hours, particularly when mechanical shaking is required. Second, if the extraction or dissolution process is

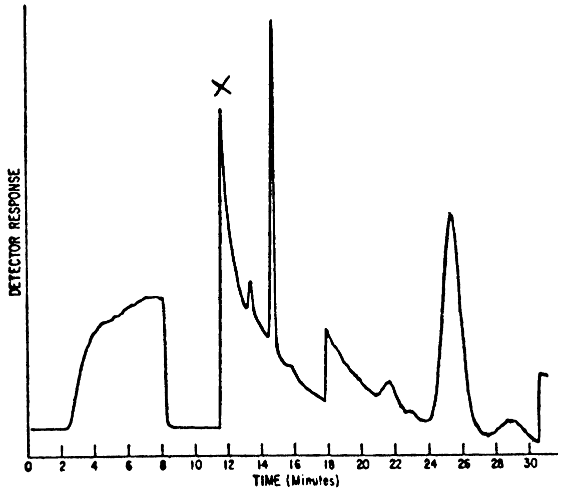

*Figure 1. FID gas chromatogram of a solution analysis of a polymer. X indicates a sensitivity change.*

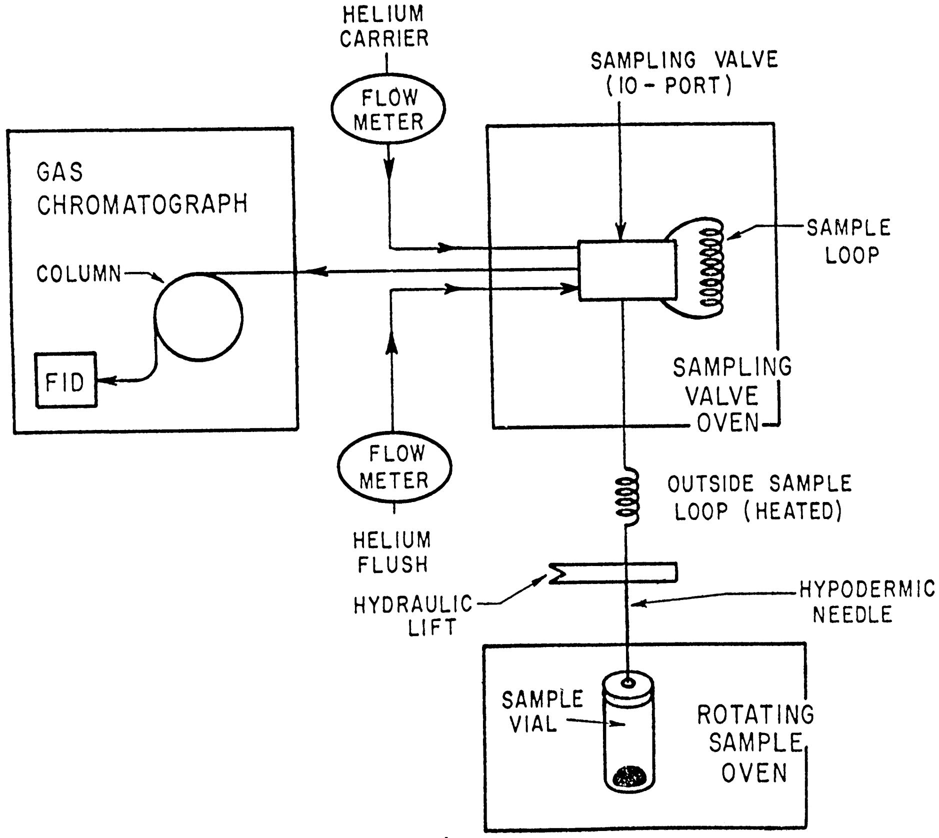

*Figure 2. Schematic diagram of automated headspace sampler with gas chromatograph.*

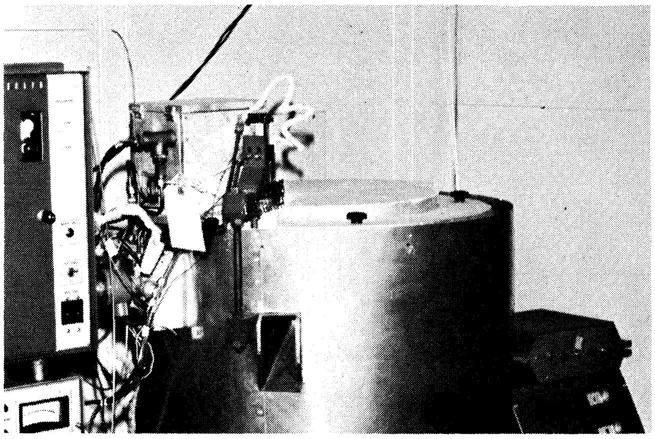

*Figure 3. Photograph of automated headspace sampler.*

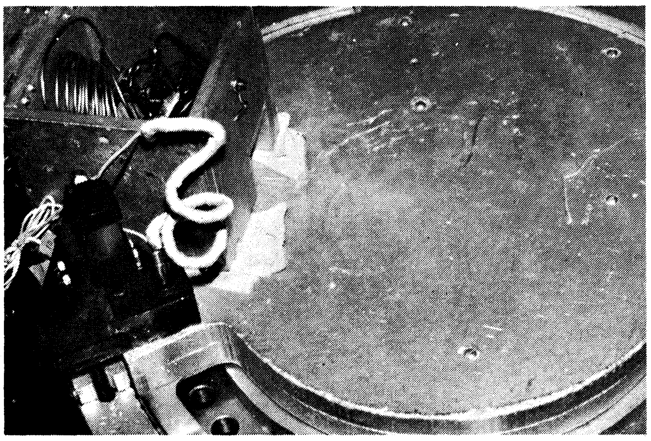

*Figure 4. Photograph of top of automated headspace sampler.*

incomplete, the residual chemicals of interest will be partitioned between two phases — the solid or swelled polymer and the liquid solution. This partition can be accounted for by proper calibration, but this is often a difficult procedure and is not often done in reality. Third, it is seldom possible to avoid getting polymer in the GC column. Build-up of polymer residue in the injection port or first few centimeters of the column will inevitably lead to degraded chromatographic performance. This difficulty can sometimes be alleviated by the use of a glass liner in the injection port, but this remedy is not entirely satisfactory. Fourth, these solutions are usually quite viscous at a typical concentration of five percent (w/v) of polymer. Therefore, syringes plug easily and usually must be cleaned after each injection.

The chromatographic aspect of the analysis also encounters some serious problems and is really the limiting factor for trace analysis. First, there is normally a large solvent peak which interferes with the analysis of trace amounts. Use of solvents that have little or no flame response (water and carbon disulfide, for example) can help, but one is restricted to those solvents that dissolve or extract the polymer satisfactorily. Second, solvent impurities may interfere. In trace analysis especially, some of the chemicals to be analysed may also appear as impurities in all but the most carefully prepared solvents. Third, extraneous peaks may be present. Extraction or dissolution almost invariably will cause some low molecular weight material in the polymer to be present in the solution to be chromatographed. Thus, the resulting complex solution may present separation problems for the chromatographer. Fourth, poor sensitivity at parts-per-million levels is usually experienced. The limit of detection for flame ionization analysis of polymer solutions or extracts is often only 10-20 ppm. Since determinations at one ppm or even sub-ppm levels are more typical today, solution or extraction methods are usually not sensitive enough to obtain the desired analytical results. Selective detectors, such as electron capture, photoionization and flame photometric, may help in selected cases, but these are not generally applicable to a wide variety of common analysis problems with residuals in polymers.

This general chromatographic problem with solution analysis is illustrated by the sample chromatogram in Figure 1. The curve consists of the solvent peak and several other peaks that are roughly in the range 10-100 ppm. The solvent peak covers the chromatogram from about 2-12 minutes. Obviously, analysis for trace components cannot be performed during this time span. After the 12-minute period small peaks can be detected, but the tail of the solvent still limits the precision of measurement that can be obtained. For analysis at one ppm or lower, this does not represent a satisfactory detection technique.

Hachenberg and Schmidt describe many applications of headspace analysis, but none where the sampling is done above a solid. Pausch and Sockis [2] have shown recently that headspace sampling above the solid polymer sample (without solvent) is a convenient way to achieve the desired sensitivity for trace analysis of volatile components. To prepare the sample, about one gram of the polymer is weighed into a glass vial which is then capped with an aluminum seal around a butyl rubber septum. The sample is then heated to a suitable temperature (usually 400-500 K) in a constant temperature bath to achieve an appropriate equilibrium distribution between the headspace and the solid phase. In practice, volatile residual chemicals in polymers generally tend to show preference for the headspace as compared to the solid polymer, thus the amount of material reaching the chromatograph via headspace injection is much greater than by solution injection. Often the enhancement of signal will be two or three orders of magnitude when comparing headspace to solution analysis [2].

**Table 1 Sequence of events for automated headspace sampling**

1. a) Pulse stepping motor 5 steps
   b) Flush hypodermic needle
2. Insert hypodermic needle into sample vial
4. Start helium flow to pressurize sample vial
4. Stop helium flow
5. Wait for equilibrium
6. Fill sample loop
7. a) Inject sample into G.C.
   b) Call computer or start recorder
8. Time out chromatographic analysis
9. Stop computer or recorder
10. Option Go to 6 or 11
11. Withdraw hypodermic needle
12. Go back to No. 1

**Table 2 Analysis of residual hydrocarbon in poly (acrylic acid)**

| | PPM-Hydrocarbon | | | |
|---|---|---|---|---|
| Sample | Headspace GC | Standard* Deviation | Extraction UV | Solution GC |
| A | 103 | 4 | 55 | 77 |
| B | 32 | 3 | 20 | 20 |
| C | 25 | 2 | 20 | 19 |
| D | 12 | 2 | 20 | 16 |
| E | 76 | 7 | 41 | 54 |
| F | 52 | 14 | — | — |
| G | 91 | 2 | 79 | 91 |
| H | 21 | 3 | 20 | 24 |
| I | 3200 | — | 1700 | 2175 |
| J | 2100 | — | 2100 | — |
| K | 21 | — | 20 | — |

*From analysis of multiple samples, not repeatability of injection from same sample.

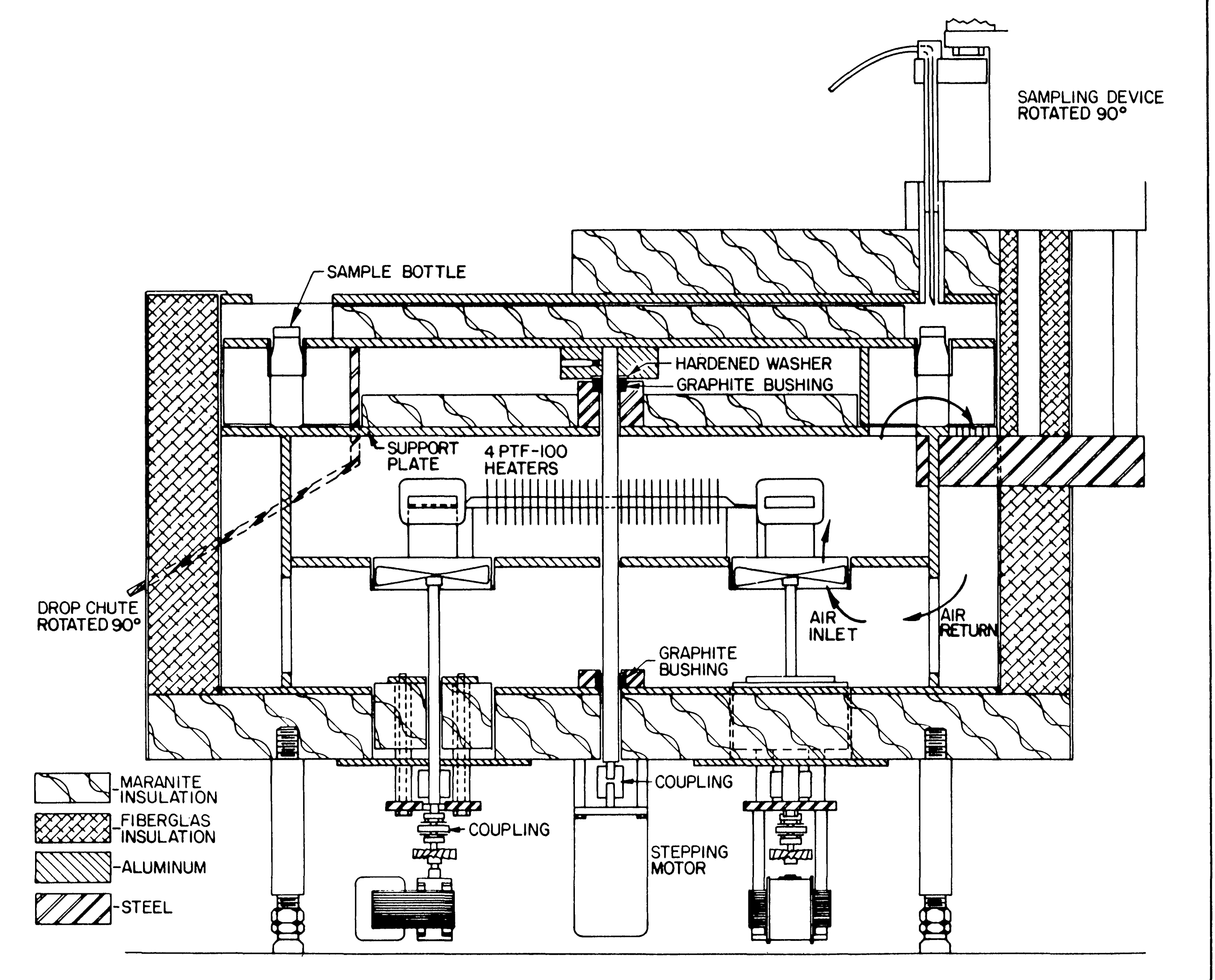

*Figure 5. Diagram of internal arrangement of oven.*

An automated commercial instrument [3] is available to perform this type of sample preparation and analysis, but it is limited to a bath temperature of 373 K or less since water is used as the heat transfer medium. However, many polymer analyses require temperatures in the 400-500 K range. Likewise, thermal desorption of adsorbents, such as charcoal, commonly uses temperatures above 500 K. This paper describes the automated system that we have built to perform these types of analyses. It is an independently controlled and operated unit which can be used with any commercial gas chromatograph. It functions solely as a means to equilibrate the sample, fill a gas sampling loop, and inject the sample into the gas chromatograph.

### Headspace sampler

Figure 2 illustrates schematically the overall arrangement for the headspace sampling system [4]. Figure 3 is a photograph of the oven and automated sampler connected to a Hewlett-Packard Model 5750 gas chromatograph. Samples are loaded through the top and there is a chute at the front where the sample bottles drop out after the samples have been analysed. The sampling valve and sample loop are located inside a heated box on the top. The solenoid valves needed to control the sampling valve and the hypodermic needle are located on the side of the unit. The two boxes at the right contain the electronics, the top one housing the digital controller and the bottom one to the power supply and sequencing logic for a stepping motor.

Figure 4 shows the top of the oven at the loading zone. It has a capacity to hold forty 20 $cm^3$ vials. The removable cover is made from Maranite. Again, the box containing the sample loop and sample valve is visible. The air cylinder used to drive the hypodermic needle into and out of the sample bottle is also visible at the left. The needle is connected to the sampling valve by a heated line. Figure 5 shows that the samples are located near the perimeter of the oven. A supporting plate for the sample bottle also acts as a baffle to prevent air from escaping the oven. In most of the oven a portion of the plate has been removed to allow air to flow around the bottles. The air exits from this area through the many small holes drilled in the outside perimeter of the support plate. There is also a partition between each sample bottle which extends vertically from the top of the type 2024 aluminum carrier down almost to the supporting plate. These partitions are needed to prevent hot air from escaping out the exit chute. In our oven we located the exit chute adjacent to the sampling station. It would have been much better to separate the two by a greater distance, thus avoiding a slight problem we had of maintaining the temperature of the sampling station.

The oven is heated by four 750 watt heaters. Two heaters are regulated by a solid state controller and two are manually switched on or off. The two controlled heaters are sufficient to maintain temperatures up to 500 K. From 500 K to 600 K, a third heater is turned on and left on. The fourth heater is necessary only for a more rapid temperature rise during initial start-up.

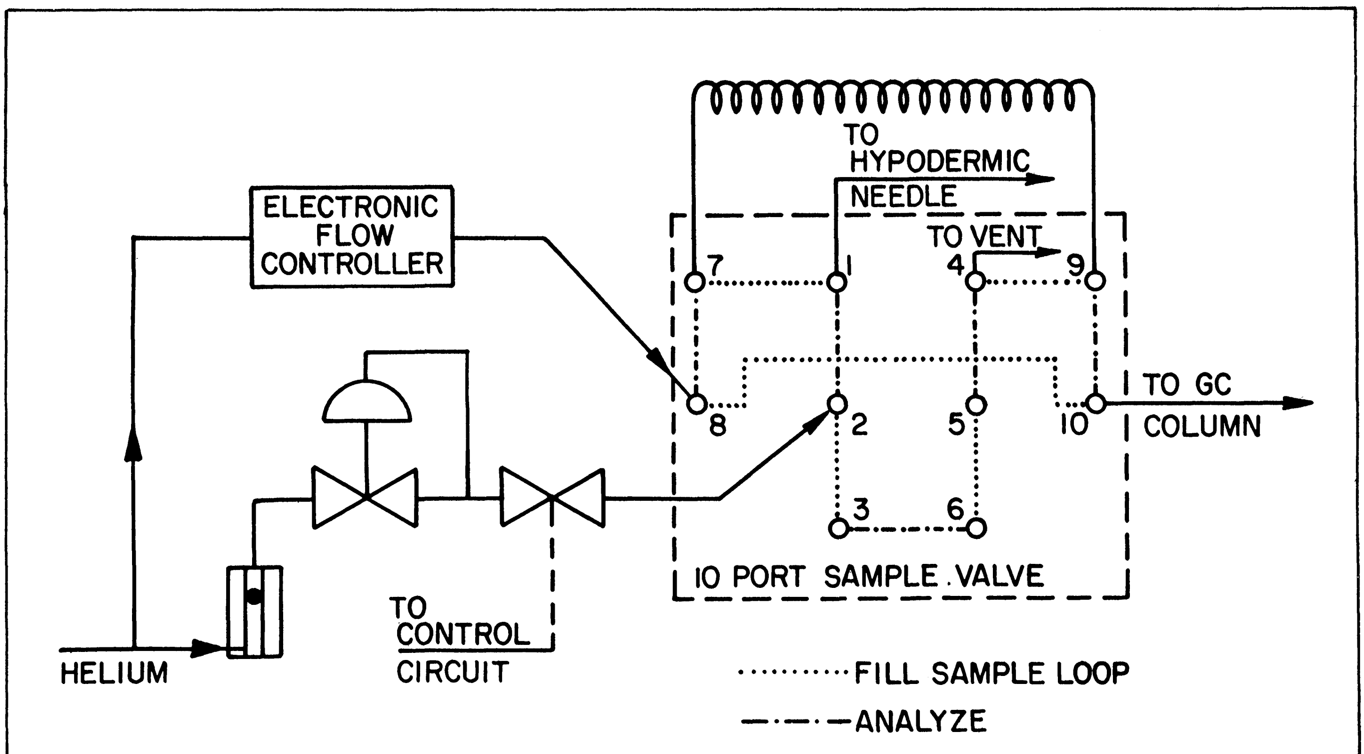

*Figure 6. Headspace sampler plumbing.*

Figure 6 also illustrates the air flow pattern. There are four air inlets, one underneath each heater which are mounted in a semi-closed compartment. A fan is also mounted at each inlet. Each fan and associated motor are a separate module which is easily removed for maintenance. Air is blown up past the heaters and exits at the top perimeter of the compartment where it flows around the samples and through the baffling on the outside perimeter of the support plate. The air is then sucked back along the outside of the oven and into the fans through one of eight holes in the side wall. Originally, the entire area on the outside perimeter of the support plate was cut out, but it was found that the air was being channeled only to the areas directly above the eight return holes. This problem was corrected by reinserting the plate and drilling many small holes in it. This gave much better control of the air flow. It also provided a way to compensate for areas with greater heat loss, such as the sampling station, by simply drilling more holes in those areas. The temperature gradient of the oven is approximately +5 K at an operating temperature of 600 K.

Figure 6 is a schematic of the plumbing arrangement for the sampling valve and its connection to the GC. The sampling valve is a 10-port, air-driven high temperature valve, manufactured by Arcas, Houston, Texas. The dashed lines show the valve in the de-energized position which is the analysis position. The dotted lines show the valve in the energized position for filling the sample loop. At the start of the sampling cycle the valve is in the analysis position. Helium is brought in through a rotameter and a flow regulator. It then goes to a solenoid valve. When the valve is open, the helium flows into port 2 of the sampling valve. When the valve is in analysis position, the flow would be from port 2 to port 1 and out to the hypodermic needle. At the time a new sample is being moved into position, the solenoid valve is opened and the needle is flushed with helium to remove any memory of the previous sample. The same path is taken to pressurize the sample bottle at a later time. Helium is also brought in through an electronic flow controller into port 8 of the sampling valve, goes to port 7, through the sample loop to port 9, on to port 10 and out to the GC column.

When the valve is energized, helium comes in port 8 and out port 10 to the GC column. The vapor sample in the bottle, which is under pressure, flows through the hypodermic needle to port 1, over to port 7 and through the loop to port 9. From port 9 it flows to port 4 where it is vented to atmospheric pressure. After the loop is filled, the valve is de-energized and the sample is swept onto the GC column by the carrier gas. Since helium flows through the sample loop for the total analysis time, the loop is well-flushed. All of these events are sequenced by the digital controller described in the next section.

### Digital controller sequence

The sequence of events to perform a headspace analysis starts after the samples have been loaded into the oven and have been heated for a time sufficient to reach equilibrium. The first sample can be moved to the position just prior to the sampling position by a manual override switch. At this point, the digital controller is switched on and the sequence of events outlined in Table II will take place automatically.

The Superior Electric Slo-Syn stepping motor produces 3 newton meters of torque and travels 1.8 degrees per step. Five steps are necessary to move a new sample bottle into position. The five pulses occur at one second intervals. The total time to reach the actual injection step (number 7) is 128 seconds. The length of the GC analysis can also be preset as required.

Figure 7 is a schematic of the electronic controller. The circuit was constructed using $T^2L$ integrated logic circuits. Its function is simply to activate and deactivate the various solenoid valve and relays, and to pulse the stepping motor at the correct time. The 60 hertz frequency is divided down to 1 hertz by IC's 3 and trigger, is used to change the sine wave into a rectangular pulse. The 60 hertz frequency, is divided down to 1 hertz by IC's 3 and 4, which are divided by 5 and divided by 12 circuits. The resulting 1 Hz pulse is then gated through IC 10 by a "chromatogram go" signal to IC's 1 and 2, which are also divided by 5 and divided by 12 circuits. The output from IC 1 has a frequency of 1/60 Hz or 1 pulse per minute. The number of times this signal pulses is then counted by a 7-bit binary counter consisting of IC 39 and 40. The maximum count is 128 minutes. The correct combination of outputs from IC 39 and IC 40 can be patched into any or all of IC's 36, 37 and 38 to generate

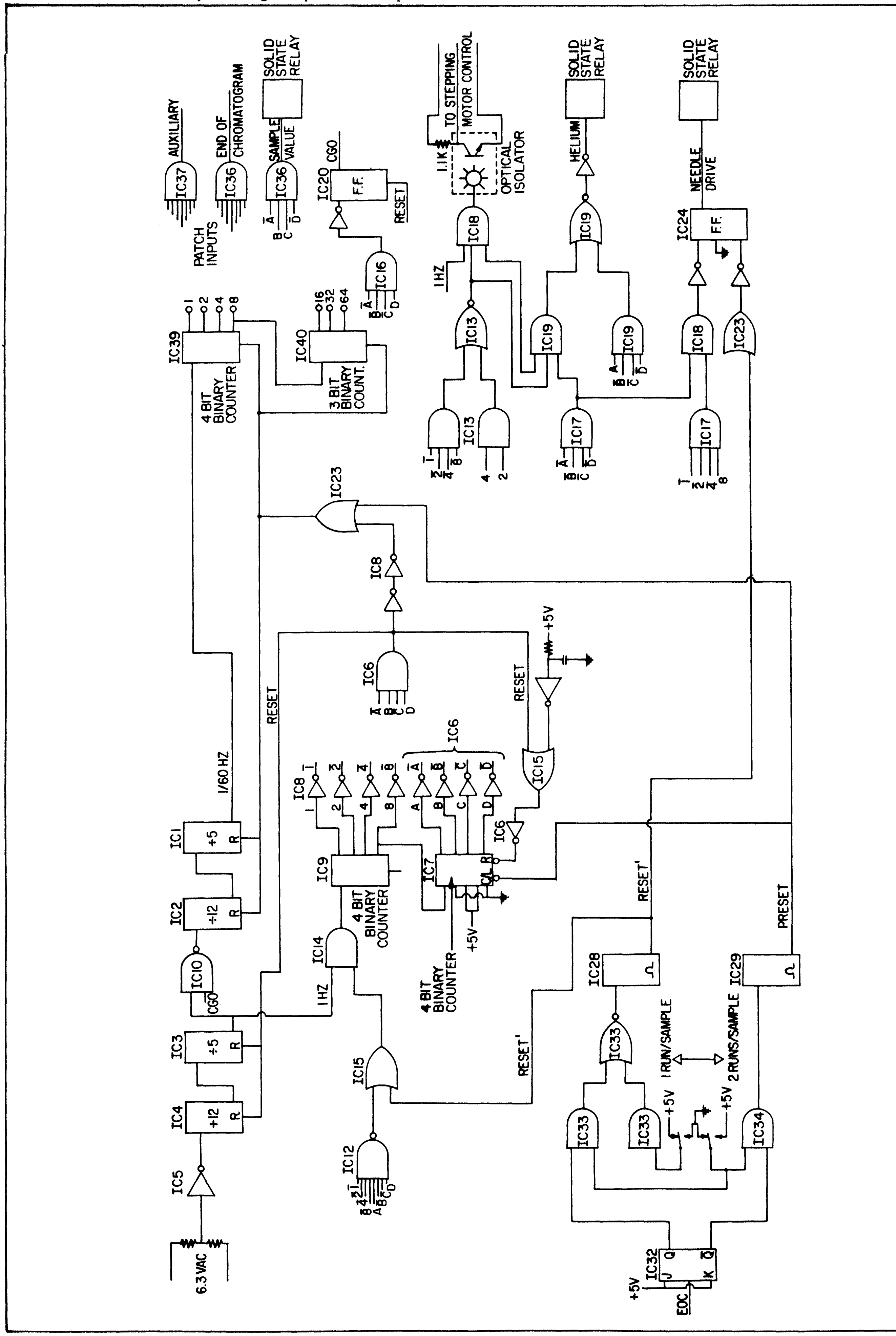

*Figure 7. Schematic of digital controller logic.*

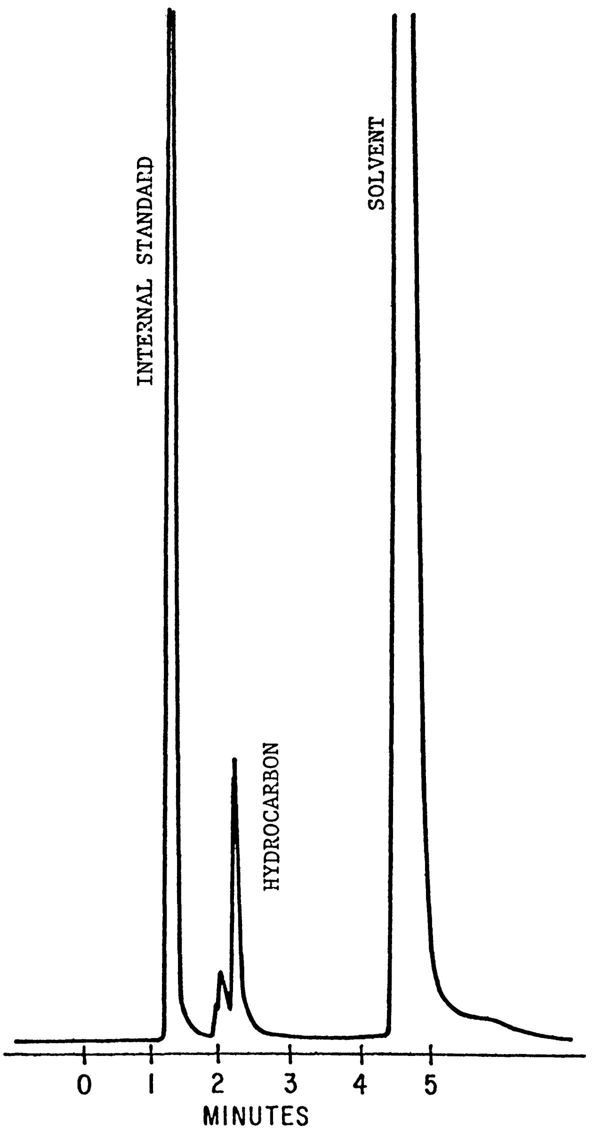

*Figure 8. Typical poly (acrylic acid) headspace chromatograph.*

output pulses at any desired time over the range of 1-128 minutes in increments of 1 minute. IC 36 is reserved for timing the duration of the chromatogram, IC's 37 and 38 are auxiliary outputs which drive solid state relays and may be used in any manner the user desires.

The 1 Hz output from IC 3 is also gated by IC 14 to an 8-bit counter consisting of IC's 9 and 7. This counter can count to 2 [8] or 256 seconds. IC 7 can be preset to any desired count. This feature is used if one wants to run 2 analyses on one sample. The outputs from this counter are decoded by various logic circuits in order to generate control signals at the correct times to perform all of the functions listed in Table 1. The timing of these functions was fixed by hardwiring and therefore cannot easily be changed.

Some of the short duration events occur prior to the start of the chromatographic run and some occur after it is finished, but none occurs during the run. Therefore, the 1 second clock is started again at the end of the chromatographic analysis time. If only one analysis per sample is desired, the 1 second clock is allowed to time out and all clocks are reset to zero, which corresponds to moving a new sample into position. For a second sampling of the same bottle, IC 7 is preset to 96 and IC's 39 and 40 are reset to zero. This procedure reverts back then to step 6 in Table 1.

### Discussion of results

The initial evaluation trials on the automated headspace sampler were performed with a poly (acrylic acid) polymer. This is a white, powdery material, and a typical chromatogram of the headspace is shown in Figure 8. An equilibration time of one hour at 125° C had previously been found sufficient to obtain an equilibrium distribution of the residual hydrocarbons between the vapor and solid phases [5]. The residual hydrocarbon for which we were analysing eluted at about 2.3 minutes; the internal standard added to the sample vial eluted at about 2.3 minutes; the internal standard added to the sample vial eluted at about 1.3 minutes; and the large peak near 5 minutes was the solvent into which the internal standard was dissolved. The small peak eluting just before the hydrocarbon is of unknown origin, but it did not interfere with the analysis.

The amount of the hydrocarbon in the polymer (even though the headspace is analysed) was determined by the method developed earlier by Pausch and Sockis [2]. The results of this analysis using our automated headspace sampler for several poly (acrylic acid) samples are given in Table 2. The reproducibility shown in column three is certainly quite acceptable for this type of analysis.

The results from two other analytical methods are also included in Table 3 to show that this sampling system is performing well. The slightly higher results in some cases compared to the solution method and the consistently higher results compared to the extraction UV procedure were not surprising.

The sampling unit has performed well so far, although we will attempt to streamline the oven somewhat in future units to reduce the bulk of the sample bottle carrier. The inertia of the present one places a heavy load on the stepping motor.

ACKNOWLEDGEMENTS

The authors wish to thank the B F Goodrich Company for support of this work. Significant contributions to this project were also provided by Dr. D. E. White, G. F. Pfeiffer, E. C. Lapka, T. D. Rice, E. G. DeCapita, R. B. Whitehead, J. J. Cangemi, and I. Sockis.

REFERENCES

[1] H. Hachenberg and A. P. Schmidt, *Gas Chromatographic Headspace Analysis,* Heyden and Son Limited, New York, 1977.
[2] J. B. Pausch and I. Sockis, *Rubber Chemistry and Technology,* **50,** 828 (1977).
[3] Perkin-Elmer Bulletin - F-42 Headspace Analyser.
[4] R. E. Arner, D. E. White, J. B. Pausch and G. F. Pfeiffer, Pittsburgh Conference 1978, Paper 278.
[5] R. P. Lattimer, J. J. Cangemi and J. B. Pausch, Pittsburgh Conference 1978, Paper 279.

